# Dual-view fusion of log-mel spectrogram encodings for dysarthria detection: use in the context of awake brain surgery

**DOI:** 10.1007/s11548-026-03743-8

**Published:** 2026-07-10

**Authors:** Nassib Abdallah, Harrison Misy, Jean-Marie Marion, Chinmayi Kanthila, Celine Panheleux, Vanessa Saliou, Romuald Seizeur, Guillaume Dardenne

**Affiliations:** 1 LaTIM UMR1101, INSERM, University of Western Brittany, Brest, France; 2 LARIS, Catholic University of the West & University of Angers, Angers, France; 3 Neurosurgery department, University Hospital of Brest, Brest, France

**Keywords:** Dysarthria, Awake brain surgery, Speech monitoring, Multimodal deep learning, Spectrograms, Gated fusion, Domain shift

## Abstract

****Purpose **:**

Intraoperative speech monitoring during awake glioma surgery is critical to detect stimulation-induced motor speech impairments such as dysarthria. However, automatic detection from short speech segments remains challenging due to limited annotations, strong acoustic variability, and transient impairments in operating-room conditions. This study investigates whether combining complementary encodings of the same log-mel representation can improve robustness under such constraints.

****Methods **:**

We propose a dual-branch EfficientNet-B0 architecture that processes two complementary views of each log-mel spectrogram: (i) a numeric amplitude-preserving representation stored as NPY, and (ii) an RGB spectrogram image stored as PNG using a fixed colormap. The resulting embeddings are fused using four strategies: naïve concatenation, attention-based fusion, and gated fusion (sigmoid and GRU-style). Models are evaluated under two settings: (i) *intra-corpus* group-wise cross-validation on TORGO (English, controlled) and DATABRASE (French, intraoperative), and (ii) *cross-corpus* transfer (TORGO→DATABRASE) to quantify domain shift. In addition, we evaluate DATABRASE with and without Demucs-based denoising to analyze the impact of operating-room noise.

****Results **:**

In intra-corpus evaluation, adaptive fusion strategies provide consistent benefits on TORGO, with gated fusion achieving the highest AUC (0.852±0.087). On raw DATABRASE, the PNG unimodal baseline yields the best mean performance (UAR 0.830±0.132; AUC 0.889±0.109), whereas denoising altered the model ranking and improved the performance of attention-based fusion (UAR 0.854±0.106). Cross-corpus testing reveals a marked degradation for all methods, confirming a severe domain shift between TORGO and intraoperative speech.

****Conclusion **:**

Dual-encoding fusion can enhance dysarthria detection when training and testing conditions are aligned and noise is controlled, but robust cross-corpus generalization remains unresolved. These results highlight both the potential and current limitations of spectrogram-based deep models for intraoperative speech monitoring, motivating future work on domain-adaptive and self-supervised learning tailored to clinical recordings.

## Introduction

Dysarthria is a motor speech disorder caused by impaired neuromuscular control, typically resulting from stroke, Parkinson’s disease, traumatic brain injury, or brain tumors [1]. In awake craniotomy for glioma resection within eloquent cortical regions [2], direct cortical stimulation (DCS) is routinely employed to map and preserve critical language and speech functions [3]. During these procedures, neurosurgeons and neuropsychologists continuously monitor the patient’s speech to detect stimulation-induced disruptions such as slurring, articulatory breakdowns, prosodic alterations, or transient speech arrest. Early identification of such anomalies is essential to preserve postoperative functional outcomes.

Despite its clinical relevance, the automatic detection of dysarthria from short speech segments remains challenging, particularly under intraoperative conditions. Compared with controlled laboratory settings, operating-room recordings exhibit limited annotations, strong acoustic variability, heterogeneous speech tasks, and transient impairments that may appear intermittently rather than as a stable condition [4]. These factors yield highly variable spectrotemporal patterns and complicate automated analysis.

Recent deep learning approaches based on spectrogram representations, including CNNs and Transformer-based architectures, have demonstrated strong performance for pathological speech classification on curated datasets [5]. Self-supervised strategies such as masked spectrogram prediction have also been proposed to mitigate data scarcity and improve robustness [6, 7]. However, generalization to intraoperative recordings remains limited due to domain shift and recording heterogeneity. In clinical practice, speech monitoring during awake surgery therefore still relies predominantly on continuous expert judgment, which is cognitively demanding and subject to inter-rater variability. Given the difficulty of collecting large annotated intraoperative datasets, we adopt a domain-adaptation setting in which models are trained on TORGO (English, controlled) and evaluated on DATABRASE (French, intraoperative).

In this context, the present study is designed to address four specific research questions. First, we investigate whether combining complementary encodings of the same log-mel spectrogram improves dysarthria detection compared with unimodal representations under controlled and clinical conditions (Q1). Second, we assess whether adaptive fusion strategies (attention and gating) provide more reliable performance than simple feature concatenation when dealing with heterogeneous and noisy speech data (Q2). Third, we analyze the stability and data sensitivity of different fusion mechanisms, with a particular focus on fold-to-fold variability in small and heterogeneous clinical cohorts (Q3). Finally, we evaluate the robustness of the proposed models under severe domain shift, by quantifying cross-corpus generalization from controlled laboratory recordings to intraoperative speech (Q4). Together, these questions aim to clarify when and how multimodal spectrotemporal fusion can meaningfully contribute to automatic speech monitoring in awake brain surgery.

## Related works

Automatic dysarthria detection has been extensively studied using spectrotemporal representations derived from short-time frequency analysis, with log-mel spectrograms emerging as a standard input due to their ability to capture articulatory imprecision, abnormal voicing, and disrupted formant trajectories [8, 9]. Early deep learning approaches primarily relied on CNNs to model local time–frequency patterns, while hybrid CNN-RNN architectures incorporating LSTM or GRU units were introduced to capture longer temporal dependencies [9]. More recently, Transformer-based models and hybrid CNN-Transformer architectures have demonstrated strong performance by dynamically reweighting informative spectrotemporal regions via self-attention [10–13].

Most studies adopt supervised learning paradigms and are evaluated on curated datasets such as TORGO [14, 15], using speaker-wise cross-validation schemes to mitigate identity leakage. Under these controlled conditions, very high classification performance has been reported, often exceeding 96-99% accuracy [16–19]. However, such near-ceiling results must be interpreted cautiously, as dysarthria is modeled as a speaker-level attribute under standardized recording conditions, which may limit robustness to transient impairments and clinical variability.

To address data scarcity, self-supervised learning strategies such as autoencoders and masked spectrogram prediction have been explored [20, 21]. Nevertheless, their application to pathological speech remains limited, and cross-corpus robustness under clinical conditions has not been systematically investigated. These limitations motivate the present work, which focuses on multimodal spectrotemporal fusion and explicitly evaluates robustness across controlled and intraoperative datasets.

## Materials and methods

### Datasets

#### TORGO corpus

TORGO is a public benchmark for dysarthric speech analysis [14, 15], with controlled recordings from 15 speakers (8 dysarthric, 7 controls). We use 1,958 short segments with binary labels derived from official speaker-level annotations, resulting in near-balanced segment counts. Because labels are speaker-determined, all evaluations rely on strict group-wise splitting to prevent identity leakage.

#### DATABRASE intraoperative corpus

DATABRASE is a clinical corpus collected during awake glioma surgery at the University Hospital of Brest [12]. Speech segments are recorded intraoperatively during direct cortical stimulation and annotated by expert clinicians for dysarthric features. The dataset includes 183 segments from 11 patients (143 non-dysarthric, 40 dysarthric) and exhibits substantial operating-room noise, patient heterogeneity, and transient impairments, making it a clinically realistic target domain for robustness and domain shift evaluation.

### Preprocessing and spectrotemporal representation

All recordings are converted to mono and resampled to 16 kHz, covering the 0–8 kHz band that contains most diagnostically relevant speech information, including formant structure, spectral slope, and voicing irregularities [22]. Each segment is trimmed using a 30 dB energy threshold to remove leading and trailing silences.

Log-mel spectrograms are computed offline using a 512-sample window (32 ms), a 256-sample hop (16 ms), and 128 mel filter banks. This configuration provides sufficient frequency resolution to capture formant-related distortions and dysarthria-specific spectral alterations [23], while maintaining adequate temporal resolution for rapid articulatory events.

Each log-mel spectrogram $S \in \mathbb {R}^{128 \times T}$ is stored using two complementary encodings:

- **NPY modality:** *S* is stored as a numeric array and normalized per sample to [0, 1]. To ensure compatibility with ImageNet-pretrained backbones, the single-channel spectrogram is replicated to form a 3-channel tensor $x_{\text {NPY}} \in \mathbb {R}^{3 \times 128 \times T}$.
- **PNG modality:** *S* is converted into an RGB image $x_{\text {PNG}}$ using a fixed colormap applied consistently across all samples.

Both modalities are resized to 224×224 pixels using bilinear interpolation prior to network input. The NPY representation preserves fine-grained log-mel energy distributions, whereas the PNG encoding emphasizes spatial patterns such as banding, temporal smearing, and disrupted harmonic structures. In addition, RGB inputs align with the inductive biases of ImageNet-pretrained convolutional encoders, facilitating transfer of generic low-level visual features to spectrogram analysis.

**Fig. 1:**
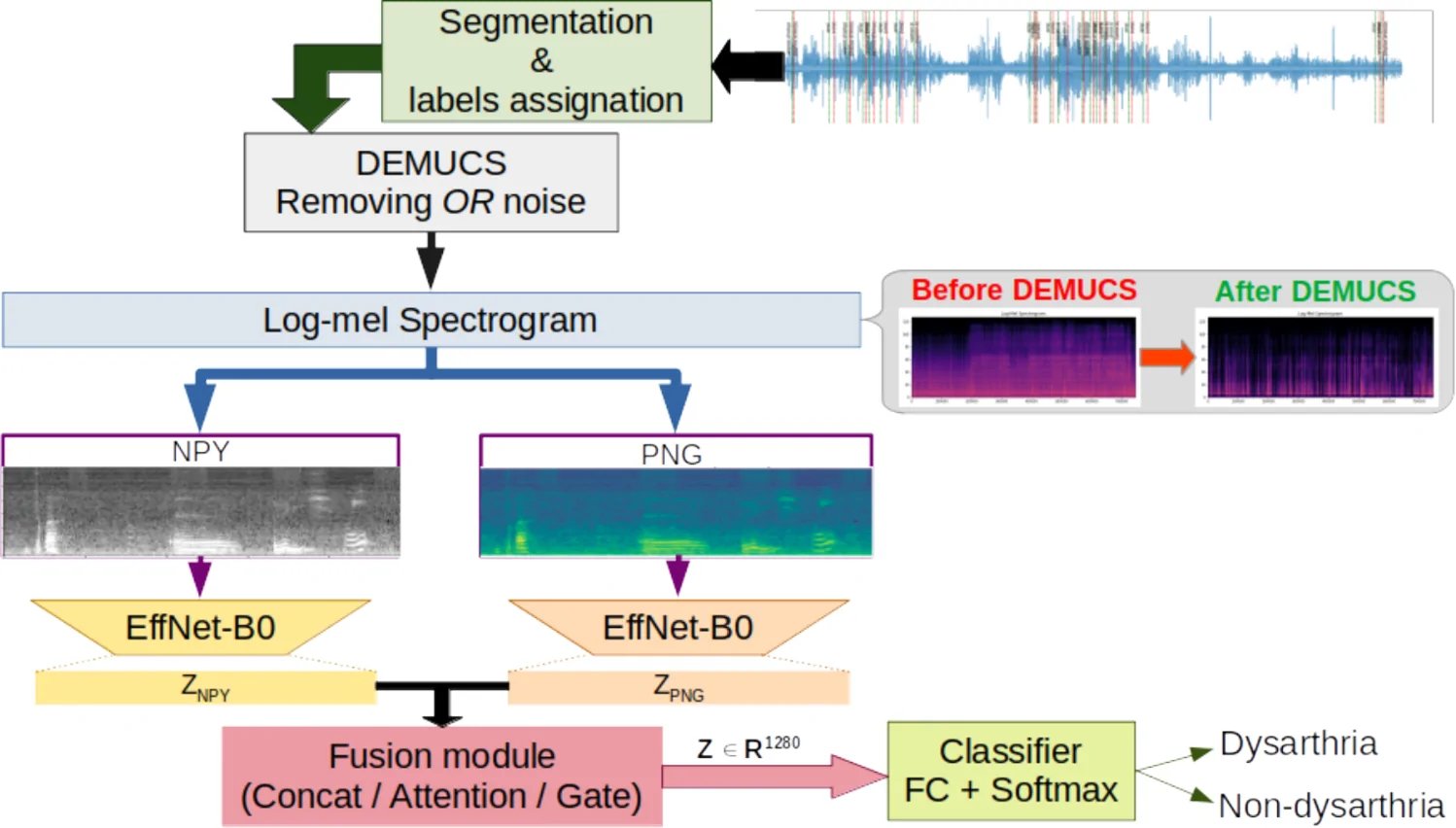
Overview of the proposed dysarthria detection pipeline

An overview of the complete processing and classification pipeline is provided in Fig. 1.

To isolate the impact of operating-room noise on dysarthria detection, we additionally evaluate all models on a denoised version of DATABRASE using Demucs [24, 25], a deep-learning-based source separation architecture. This step is applied *only* to DATABRASE because it was acquired intraoperatively and contains substantial non-stationary background noise (e.g., medical equipment, ventilation systems, staff movement, and room reverberation) that is largely absent from the controlled recording conditions of TORGO. In this work, we quantify to what extent classification errors stem from external acoustic interference versus intrinsic speech-motor alterations. Denoising was applied to DATABRASE at the waveform level prior to log-mel extraction, yielding a denoised version of each segment while preserving the original temporal boundaries.

### Dual-branch efficientNet backbone

We adopt a dual-branch architecture in which each modality is processed by a dedicated EfficientNet-B0 encoder. EfficientNet-B0 provides a favorable trade-off between accuracy, parameter count, and inference speed, and benefits from ImageNet pretraining. Let $x_{\text {NPY}}$ and $x_{\text {PNG}}$ denote the NPY and PNG inputs, respectively. Each branch produces a 1280-dimensional embedding:

1 $$\begin{aligned} f_{\text {NPY}}&= E_{\text {NPY}}(x_{\text {NPY}}) \in \mathbb {R}^{1280}, \end{aligned}$$

2 $$\begin{aligned} f_{\text {PNG}}&= E_{\text {PNG}}(x_{\text {PNG}}) \in \mathbb {R}^{1280}, \end{aligned}$$

where $E_{\text {NPY}}$ and $E_{\text {PNG}}$ share the same EfficientNet-B0 architecture but do not share weights.

This design enables each encoder to specialize to its input representation and allows the RGB-encoded spectrogram to leverage ImageNet-pretrained EfficientNet features, which may capture higher-level spatial patterns complementary to the fine-grained amplitude information preserved in the numeric NPY representation.

### Fusion strategies

We investigate a representative and well-established set of fusion families, chosen to cover complementary levels of adaptivity while remaining compatible with limited clinical data. Although many alternative fusion mechanisms exist, we focused on canonical strategies commonly used in multimodal deep learning: (i) non-adaptive embedding concatenation, (ii) modality-wise adaptive fusion via soft attention, and (iii) feature-wise adaptive fusion via gating mechanisms. In addition, a GRU-inspired gated variant is evaluated to assess whether a more expressive nonlinear interaction further improves performance.

#### Naïve concatenation

As a baseline, we perform feature concatenation at the embedding level:

3 $$\begin{aligned} z_{\text {concat}} = [f_{\text {NPY}}; f_{\text {PNG}}] \in \mathbb {R}^{2560}. \end{aligned}$$

In our implementation, $z_{\text {concat}}$ is directly regularized with dropout (rate 0.3) and fed to a single linear layer producing logits for the two classes:

4 $$\begin{aligned} \ell = \textbf{W} z_{\text {concat}} + \textbf{b} \in \mathbb {R}^{2}, \end{aligned}$$

followed by a softmax to obtain class probabilities. This strategy is simple and widely used in multimodal learning. However, it assigns fixed contributions to both modalities and cannot be adapted to sample-wise modality reliability.

#### Attention-based fusion

To introduce data-dependent weighting, we implement soft attention over the two embeddings. Attention scores are obtained via:

5 $$\begin{aligned} w_{\text {NPY}}&= \textbf{u}_2^\top \tanh (\textbf{W}_1 f_{\text {NPY}}) + c_2, \end{aligned}$$

6 $$\begin{aligned} w_{\text {PNG}}&= \textbf{u}_4^\top \tanh (\textbf{W}_3 f_{\text {PNG}}) + c_4 \end{aligned}$$

with $\textbf{W}_1,\textbf{W}_3 \in \mathbb {R}^{1280 \times 1280}$, $\textbf{u}_2,\textbf{u}_4 \in \mathbb {R}^{1280}$ and scalar biases $c_2,c_4 \in \mathbb {R}$. The tanh activation stabilizes training by bounding the intermediate activations.

The scalar weights are normalized via a softmax:

7 $$\begin{aligned} \alpha _{\text {NPY}}&= \frac{\exp (w_{\text {NPY}})}{\exp (w_{\text {NPY}}) + \exp (w_{\text {PNG}})}, \end{aligned}$$

8 $$\begin{aligned} \alpha _{\text {PNG}}&= 1 - \alpha _{\text {NPY}}. \end{aligned}$$

The fused representation is then computed as a convex combination:

9 $$\begin{aligned} z_{\text {att}} = \alpha _{\text {NPY}} f_{\text {NPY}} + \alpha _{\text {PNG}} f_{\text {PNG}}. \end{aligned}$$

In our implementation, $z_{\text {att}}$ is regularized with dropout (rate 0.3) and passed to a single linear layer that outputs logits for binary classification, followed by a softmax:

10 $$\begin{aligned} \ell = \textbf{W} z_{\text {att}} + \mathbf {(c2,c4)^\top } \in \mathbb {R}^{2}. \end{aligned}$$

#### Sigmoid gated fusion

Gated fusion introduces feature-wise interpolation between modalities. The two embeddings are concatenated:

11 $$\begin{aligned} f_{\text {cat}} = [f_{\text {NPY}}; f_{\text {PNG}}] \in \mathbb {R}^{2560}, \end{aligned}$$

and passed through a gating module:

12 $$\begin{aligned} g = \sigma \left( \textbf{W}_2 \, \text {ReLU}(\textbf{W}_1 f_{\text {cat}} + \textbf{b}_1) + \textbf{b}_2\right) \in \mathbb {R}^{1280}, \end{aligned}$$

where $\textbf{W}_1 \in \mathbb {R}^{1280 \times 2560}$ and $\textbf{W}_2 \in \mathbb {R}^{1280 \times 1280}$, with corresponding bias terms; $\sigma (\cdot )$ denotes the sigmoid function. The fused representation is

13 $$\begin{aligned} z_{\text {gate}} = g \odot f_{\text {NPY}} + (1 - g) \odot f_{\text {PNG}}. \end{aligned}$$

This feature-level gating acts as a content-aware mixture-of-experts, enabling the model to select, for each latent dimension, which modality should dominate.

#### GRU-style gated fusion

We further explore a GRU-inspired fusion, with reset "r" and update gates "z":

14 $$\begin{aligned} z&= \sigma (\textbf{W}_z f_{\text {cat}} + \textbf{b}_z), \end{aligned}$$

15 $$\begin{aligned} r&= \sigma (\textbf{W}_r f_{\text {cat}} + \textbf{b}_r), \end{aligned}$$

where $\textbf{W}_z,\textbf{W}_r,\textbf{W}_h \in \mathbb {R}^{1280 \times 2560}$, with corresponding bias terms. The reset gate modulates the NPY embedding:

16 $$\begin{aligned} f_{\text {gate}} = [r \odot f_{\text {NPY}}; f_{\text {PNG}}], \end{aligned}$$

which is transformed into a candidate representation:

17 $$\begin{aligned} \tilde{h} = \tanh (\textbf{W}_h f_{\text {gate}} + \textbf{b}_h). \end{aligned}$$

The final fused vector is

18 $$\begin{aligned} h = (1 - z) \odot f_{\text {NPY}} + z \odot \tilde{h}. \end{aligned}$$

This formulation allows the model to preserve or overwrite NPY features depending on the sample, capturing richer interactions between modalities.

**Table 1:** Intra-corpus (INTRA) results reported as mean ± standard deviation across 5 group-stratified folds.

Dataset	Model	Accuracy	UAR	Precision	Recall	F1	AUC
TORGO	NPY	0.574 ± 0.107	0.648 ± 0.057	0.589 ± 0.259	**0.839 ± 0.235**	0.630 ± 0.140	0.769 ± 0.142
	PNG	0.649 ± 0.207	**0.716 ± 0.132**	0.688 ± 0.264	0.736 ± 0.301	0.639 ± 0.206	0.788 ± 0.152
	Concat	0.635 ± 0.176	0.699 ± 0.096	0.672 ± 0.254	0.728 ± 0.298	0.625 ± 0.181	0.801 ± 0.118
	Attention	0.662 ± 0.201	0.713 ± 0.129	0.692 ± 0.269	0.690 ± 0.297	0.629 ± 0.222	0.784 ± 0.124
	Gated (sigmoid)	0.642 ± 0.166	0.704 ± 0.096	0.666 ± 0.248	0.755 ± 0.272	0.644 ± 0.157	**0.852 ± 0.087**
	Gated (GRU)	0.644 ± 0.159	0.701 ± 0.084	0.665 ± 0.241	0.712 ± 0.284	0.625 ± 0.174	0.824 ± 0.117
DATABRASE (raw)	NPY	0.706 ± 0.209	0.708 ± 0.272	0.500 ± 0.332	0.790 ± 0.333	0.547 ± 0.365	0.710 ± 0.391
	PNG	**0.767 ± 0.236**	**0.830 ± 0.132**	**0.685 ± 0.404**	0.829 ± 0.186	**0.656 ± 0.364**	**0.889 ± 0.109**
	Concat	0.704 ± 0.310	0.658 ± 0.357	0.511 ± 0.430	0.610 ± 0.437	0.540 ± 0.419	0.688 ± 0.413
	Attention	0.642 ± 0.225	0.630 ± 0.248	0.458 ± 0.322	0.714 ± 0.416	0.535 ± 0.332	0.665 ± 0.276
	Gated (sigmoid)	0.619 ± 0.250	0.682 ± 0.221	0.471 ± 0.442	0.629 ± 0.412	0.437 ± 0.385	0.750 ± 0.388
	Gated (GRU)	0.578 ± 0.370	0.719 ± 0.195	0.626 ± 0.441	0.829 ± 0.235	0.571 ± 0.334	0.779 ± 0.358
DATABRASE (Demucs)	NPY	0.655 ± 0.231	0.676 ± 0.304	0.557 ± 0.361	0.629 ± 0.458	0.527 ± 0.322	0.742 ± 0.286
	PNG	0.577 ± 0.188	0.698 ± 0.161	0.460 ± 0.384	0.757 ± 0.301	0.427 ± 0.261	0.716 ± 0.242
	Concat	0.647 ± 0.216	0.632 ± 0.229	0.486 ± 0.351	0.629 ± 0.412	0.508 ± 0.332	0.687 ± 0.280
	Attention	**0.790 ± 0.145**	**0.854 ± 0.106**	0.621 ± 0.415	**0.914 ± 0.128**	**0.651 ± 0.357**	**0.819 ± 0.108**
	Gated (sigmoid)	0.604 ± 0.325	0.743 ± 0.212	0.561 ± 0.422	0.857 ± 0.247	0.548 ± 0.362	0.664 ± 0.313
	Gated (GRU)	0.703 ± 0.186	0.659 ± 0.294	0.506 ± 0.286	0.657 ± 0.412	0.562 ± 0.322	0.730 ± 0.265

Best UAR and best AUC within each dataset are highlighted in bold

**Table 2:** Intra-corpus (INTRA) results reported as median ± IQR across folds to emphasize robustness to fold-to-fold variability and potential outliers.

Dataset	Model	Accuracy	UAR	Precision	Recall	F1	AUC
TORGO	NPY	0.519 ±0.172	0.682 ±0.080	0.588 ±0.210	**0.955 ±0.112**	0.605 ±0.132	0.788 ±0.220
	PNG	**0.656 ±0.157**	0.694 ±0.086	0.698 ±0.436	0.763 ±0.315	0.642 ±0.183	0.807 ±0.104
	Concat	0.627 ±0.162	0.672 ±0.086	**0.741 ±0.326**	0.754 ±0.371	0.645 ±0.194	0.796 ±0.092
	Attention	0.670 ±0.082	0.670 ±0.074	0.665 ±0.401	0.739 ±0.373	**0.667 ±0.264**	0.765 ±0.071
	Gated (sigmoid)	0.683 ±0.196	**0.705 ±0.041**	0.681 ±0.344	0.746 ±0.280	0.622 ±0.177	**0.847 ±0.059**
	Gated (GRU)	0.648 ±0.016	0.678 ±0.052	0.620 ±0.254	0.712 ±0.321	0.622 ±0.197	0.792 ±0.172
DATABRASE (raw)	NPY	0.594 ±0.346	0.783 ±0.458	0.625 ±0.373	**1.000 ±0.286**	0.667 ±0.537	0.767 ±0.263
	PNG	0.800 ±0.191	**0.857 ±0.226**	0.800 ±0.400	0.857 ±0.286	**0.833 ±0.304**	0.865 ±0.130
	Concat	**0.833 ±0.290**	0.812 ±0.629	0.571 ±0.684	0.714 ±0.667	0.727 ±0.563	**0.961 ±0.683**
	Attention	0.667 ±0.243	0.675 ±0.262	0.556 ±0.267	0.857 ±0.286	0.625 ±0.261	0.701 ±0.060
	Gated (sigmoid)	0.700 ±0.229	0.695 ±0.161	0.667 ±0.645	0.571 ±0.429	0.615 ±0.685	0.850 ±0.169
	Gated (GRU)	0.700 ±0.500	0.786 ±0.261	**0.800 ±0.687**	**1.000 ±0.429**	0.727 ±0.250	**0.974 ±0.247**
DATABRASE (Demucs)	NPY	0.722 ±0.290	0.747 ±0.222	0.600 ±0.148	0.857 ±0.714	0.683 ±0.261	**0.831 ±0.128**
	PNG	0.508 ±0.260	0.750 ±0.149	0.400 ±0.467	0.857 ±0.357	0.444 ±0.266	0.817 ±0.307
	Concat	0.656 ±0.067	0.619 ±0.066	0.571 ±0.541	0.571 ±0.429	0.571 ±0.255	0.714 ±0.069
	Attention	**0.800 ±0.128**	**0.857 ±0.014**	0.700 ±0.647	**1.000 ±0.143**	**0.824 ±0.333**	0.810 ±0.074
	Gated (sigmoid)	0.600 ±0.528	0.714 ±0.234	**0.778 ±0.597**	**1.000 ±0.143**	0.600 ±0.546	0.714 ±0.568
	Gated (GRU)	0.722 ±0.355	0.721 ±0.475	0.625 ±0.095	0.714 ±0.429	0.667 ±0.112	0.818 ±0.400

Best UAR and best AUC within each dataset are highlighted in bold

### Classifier and training procedure

For unimodal baselines, a single EfficientNet-B0 encoder followed by a linear classification head predicts the dysarthria label from either $x_{\textrm{NPY}}$ or $x_{\textrm{PNG}}$. For multimodal models, the fused representation (Sect. Fusion strategies) is passed through a dropout layer (rate 0.3) and a final linear layer to produce logits for binary classification. Model parameters are optimized using a weighted cross-entropy loss:

19 $$\begin{aligned} \mathcal {L} = - w_{y}\,\log \left( \text {softmax}(\ell )_y\right) , \end{aligned}$$

where ℓ denotes the output logits, $y \in \{0,1\}$ is the ground-truth label, and $w_y$ is the class weight computed from the training split of each fold to mitigate class imbalance. In practice, we set $w_0=1$ and $w_1 = n_0/n_1$, where $n_0$ and $n_1$ are the numbers of non-dysarthric and dysarthric training samples, respectively.

***Intra-corpus evaluation (INTRA)***

We report intra-corpus performance using *K*-fold cross-validation with *group-wise* partitioning to prevent identity leakage. Groups correspond to speaker/patient identities (derived from the segment identifier), and folds are constructed such that no group appears in both training and test sets. To approximately preserve class balance at the *group* level, we perform a stratified split over groups using a single label per group. For datasets in which groups are label-pure (e.g., TORGO, where dysarthria is defined at the speaker level), the group label is defined as the majority label within the group. Within each fold, we further create a validation set from the training groups using a group-respecting shuffle split (15% of the training data) for early stopping. This evaluation protocol is deliberately conservative and prioritizes generalization across speakers or patients rather than segment-level memorization.

***Cross-corpus evaluation (INTER)***

To evaluate robustness to domain shift, models are trained on TORGO and tested on DATABRASE (raw or Demucs-denoised). The TORGO training set is split into training and validation partitions using the same group-respecting procedure, while all DATABRASE segments are used as an external test set.

**Table 3:** Cross-corpus (INTER) evaluation: models trained on TORGO and tested on DATABRASE (raw or Demucs-denoised).

Test set	Model	Accuracy	UAR	Precision	Recall	F1	AUC
DATABRASE (raw)	NPY	0.290	**0**.**509**	**0**.**222**	**0**.**900**	**0**.**356**	0.488
	PNG	**0**.**350**	0.323	0.109	0.275	0.156	0.302
	Concat	0.268	0.396	0.174	0.625	0.272	**0**.**492**
	Attention	0.328	0.345	0.133	0.375	0.196	0.325
	Gated (sigmoid)	0.268	0.342	0.144	0.475	0.221	0.385
	Gated (GRU)	0.317	0.374	0.154	0.475	0.233	0.419
DATABRASE (Demucs)	NPY	0.205	0.452	0.172	0.848	0.286	0.460
	PNG	0.222	0.404	0.153	0.697	0.251	0.315
	Concat	0.205	**0**.**476**	**0**.**180**	**0**.**909**	**0**.**300**	0.524
	Attention	0.210	0.444	0.169	0.818	0.280	0.456
	Gated (sigmoid)	0.222	0.439	0.167	0.788	0.275	0.487
	Gated (GRU)	**0**.**233**	0.470	0.177	0.848	0.293	**0**.**583**

Best results per metric within each test set are highlighted in bold

**Fig. 2:**
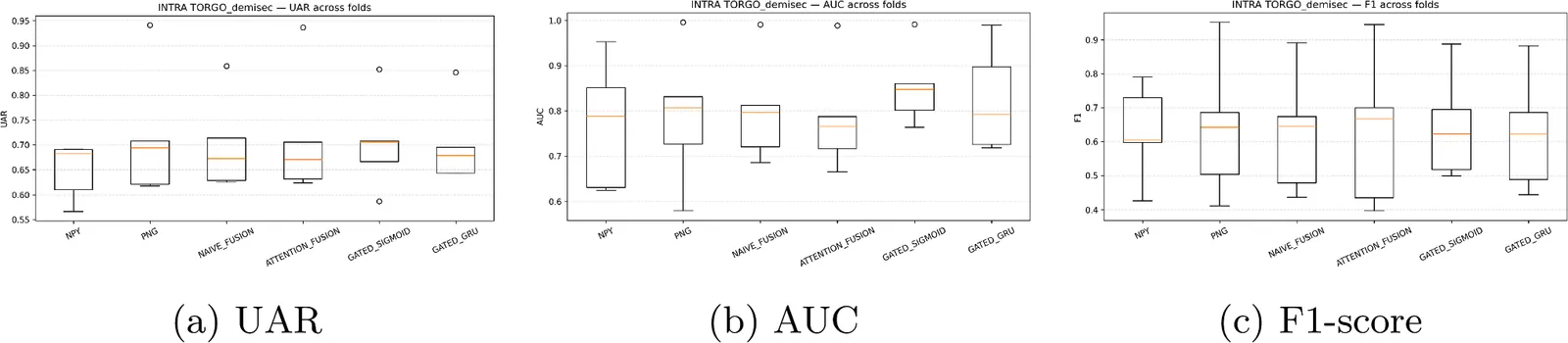
Inter-fold variability on TORGO (INTRA)

**Fig. 3:**
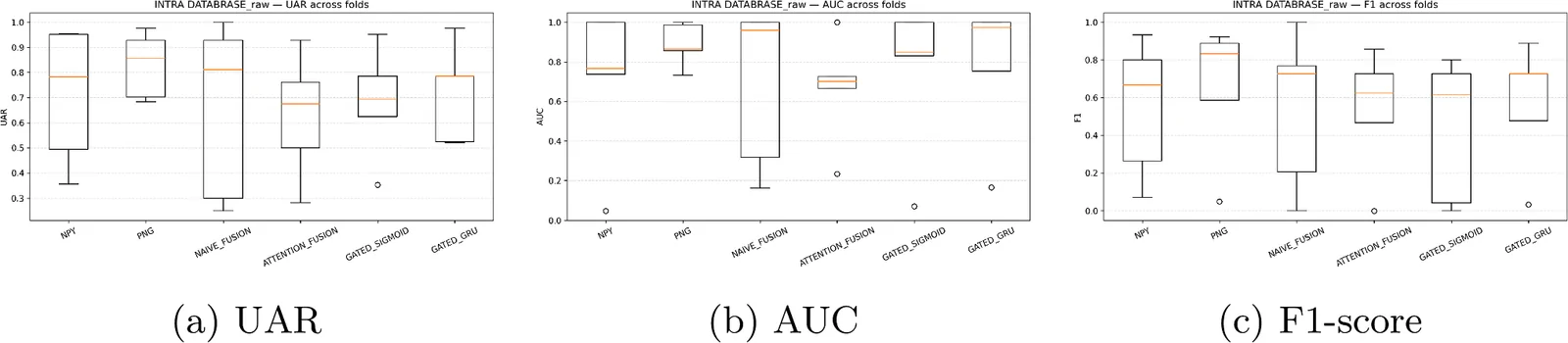
Inter-fold variability on DATABRASE (raw)

**Fig. 4:**
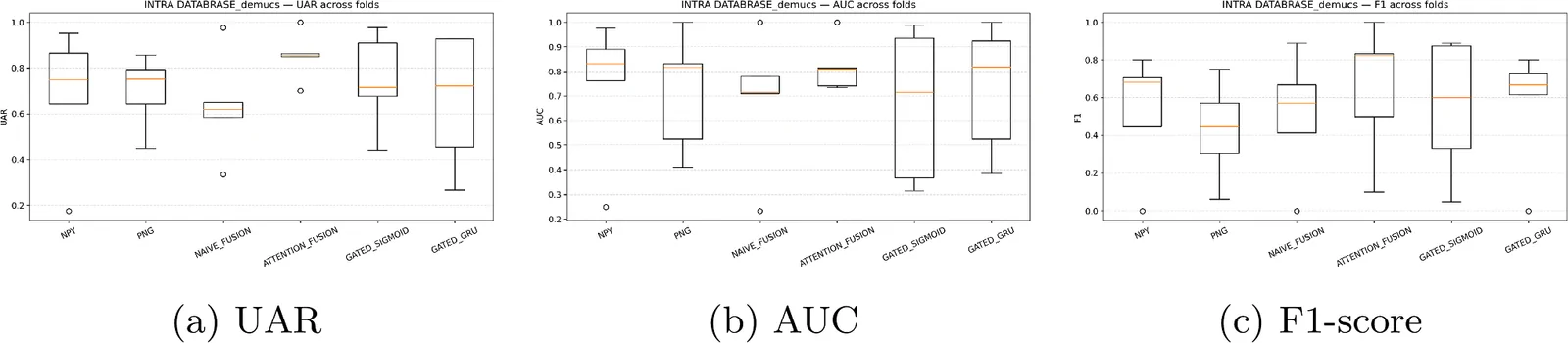
Effect of denoising on inter-fold variability (DATABRASE INTRA)

***Optimization settings***

Models are trained with Adam optimizer (learning rate $10^{-4}$, batch size 16) for up to 30 epochs with early stopping (patience 5, best validation loss). Performance is evaluated on held-out test folds using accuracy, unweighted average recall (UAR; i.e. balanced accuracy), F1-score, and area under the ROC curve (AUC).

## Results

All experiments are evaluated in two settings: intra-corpus (INTRA) group-stratified cross-validation and cross-corpus (INTER) transfer from TORGO to DATABRASE. Given the small number of patients and heterogeneous intraoperative conditions in DATABRASE, we report both mean±SD and median±IQR across folds to characterize variability and robustness.

### INTRA performance across datasets

On TORGO, fusion provides consistent gains over unimodal baselines, with gated fusion reaching the best AUC (0.852±0.087; Table 1). On raw DATABRASE, the PNG-based unimodal model achieved the highest mean UAR (0.830±0.132) and AUC (0.889±0.109), while variability remains substantial across patient-wise folds (Table 2). After Demucs denoising, attention-based fusion achieved the best UAR performance (UAR 0.854±0.106; Table 1).

### INTER cross-corpus robustness under domain shift

Cross-corpus transfer from TORGO to DATABRASE yields a marked performance degradation for all methods (Table 3), confirming a severe domain shift between controlled English recordings and intraoperative French speech. Models frequently achieve high dysarthria recall at the expense of precision, indicating a tendency to over-predict dysarthria under mismatch conditions. Demucs denoising provides only limited gains (e.g., improved AUC for gated GRU) and does not close the generalization gap, motivating domain-robust learning beyond supervised training on TORGO alone.

**Fig. 5:**
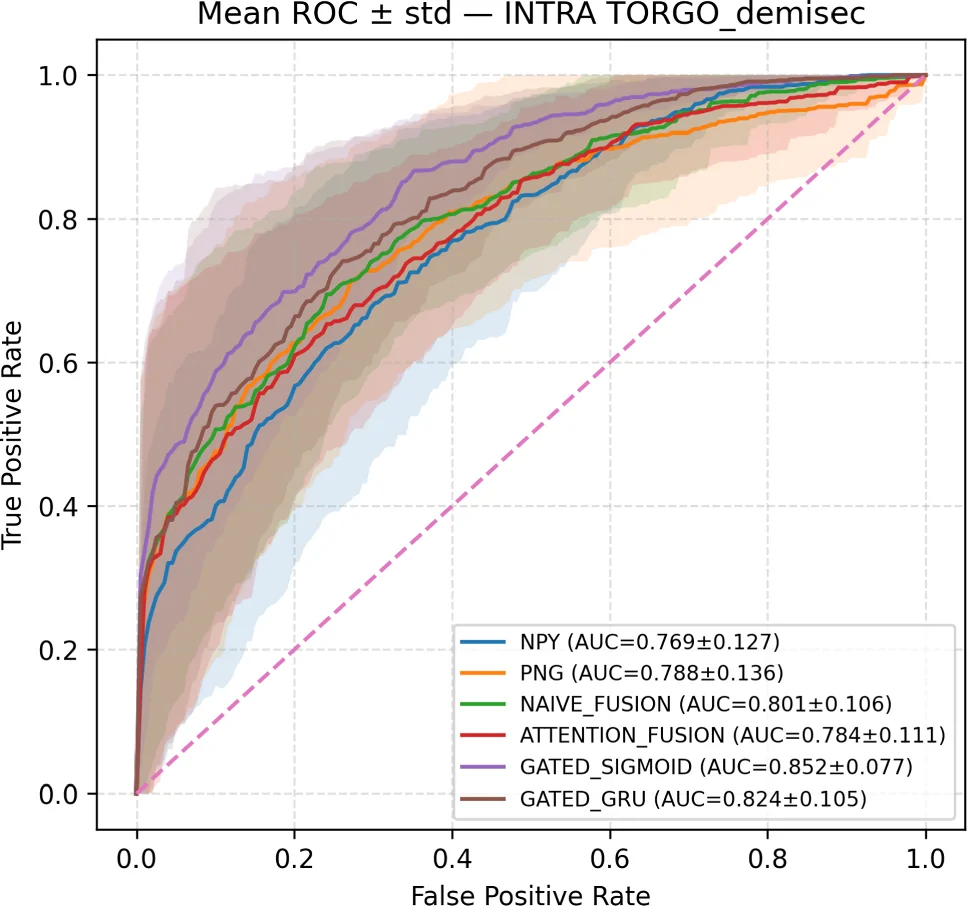
Mean ROC ± standard deviation on TORGO (INTRA)

## Discussion

**Fig. 6:**
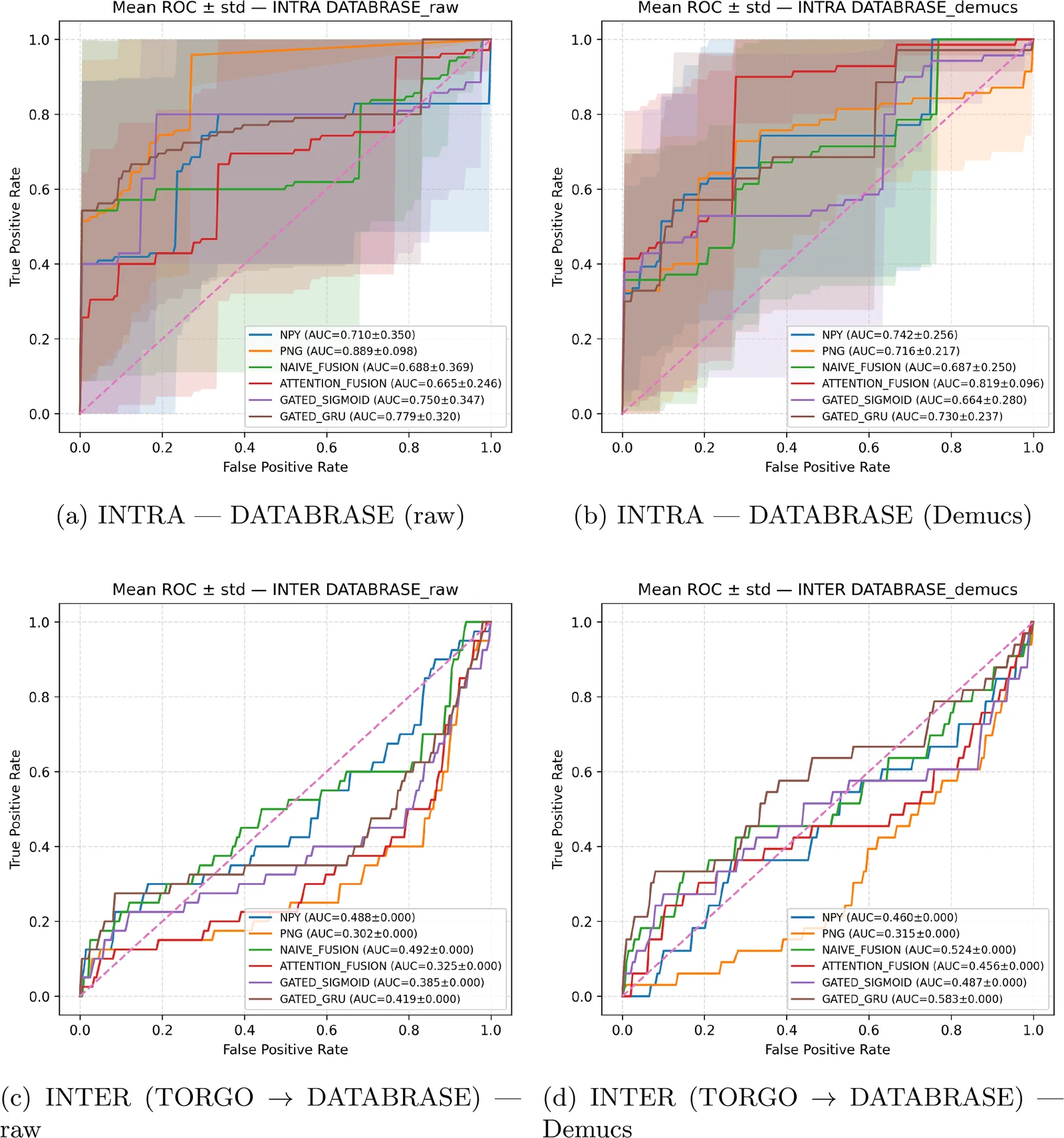
ROC curves with variability across folds (INTRA) and cross-corpus transfer (INTER)

This study assessed whether fusing two complementary encodings of the same log-mel spectrogram (NPY amplitude view vs. PNG colormapped view) improves dysarthria detection, and whether such gains persist under intraoperative noise and cross-corpus domain shift. Across TORGO and DATABRASE, we compared non-adaptive (concatenation) and adaptive (attention, gating) fusion and explicitly examined stability through fold-level variability (Figs. 2, 3, 4, 5, and 6).

### Q1 – When does dual-encoding fusion help?

On TORGO (INTRA), multimodal fusion improves discrimination over unimodal inputs (Table 1), with gated sigmoid reaching the best AUC (0.852±0.087) versus NPY (0.769±0.142) and PNG (0.788±0.152), consistent with stable ROC profiles across folds (Fig. 5). On raw DATABRASE, the PNG unimodal baseline achieves the strongest mean performance (UAR 0.830±0.132; AUC 0.889±0.109), suggesting that ImageNet-pretrained vision features can be particularly competitive in low-data, noisy clinical settings. After Demucs denoising, the ranking shifts and attention-based fusion achieved the best UAR performance (UAR 0.854±0.106), with improved ROC behavior (Fig. 6b).

### Q2 – Are adaptive fusion mechanisms necessary beyond concatenation?

Across datasets, naïve concatenation rarely achieved the best performance and shows higher sensitivity to fold composition (Tables 1 and 2). In contrast, adaptive fusion mechanisms consistently rank among the top performers: attention is most reliable on denoised DATABRASE (highest UAR/F1 with reduced dispersion; Fig. 4), whereas gated fusion achieves the strongest TORGO discrimination (highest AUC; Table 1). These results support the need for sample-dependent modality weighting when modality reliability varies across noise and recording conditions.

### Q3 – What is the trade-off of gating: adaptivity vs. data sensitivity?

Gating offers feature-wise, sample-specific interpolation between embeddings and is effective under controlled conditions (TORGO), where it yields high AUC with limited fold-to-fold variability (Table 1, Fig. 2). However, on DATABRASE gating is less stable, especially in the raw setting, where large SD/IQR values indicate strong dependence on patient split composition (e.g., gated GRU AUC 0.779±0.358; Table 1). This behavior suggests that gating mechanisms are more sensitive to limited sample size and patient heterogeneity than attention-based fusion.

### Q4 – Does fusion mitigate cross-corpus domain shift?

In INTER evaluation (TORGO→DATABRASE), all models exhibited substantial performance degradation (Table 3, Fig. 6), indicating that multimodal fusion alone does not resolve the mismatch between controlled English recordings and intraoperative French speech. The consistent pattern of high recall but low precision suggests systematic over-detection of dysarthria under mismatch conditions, likely driven by language/phonetic differences, recording setups, and label mismatch (speaker-level pathology vs. transient intraoperative impairment). Demucs denoising provided only modest gains (e.g., improved AUC for gated GRU) and does not close the generalization gap, motivating domain-robust learning strategies (domain adaptation and/or self-supervised pretraining) tailored to clinical audio.

### Inter-fold variability reflects intrinsic clinical heterogeneity

Although average performance provides a global summary, fold-wise analysis reveals marked differences between controlled and intraoperative conditions. On TORGO, inter-fold dispersion remains limited, consistent with standardized recordings and speaker-level labels (Fig. 2). In contrast, DATABRASE exhibits substantial variability across patient-wise folds (Fig. 3), indicating that performance is highly sensitive to patient composition and intraoperative context. This variability reflects the transient and task-dependent nature of speech impairments during awake surgery rather than optimization instability. Demucs denoising partially reduces dispersion for several models, particularly attention-based fusion (Fig. 4), suggesting that non-stationary operating-room noise contributes to fold-specific failures. These results highlight the importance of reporting stability and worst-case behavior, in addition to mean performance, when targeting intraoperative speech monitoring.

### Limitations and future directions

This study is limited by the small number of patients and class imbalance in DATABRASE, which induces substantial fold-to-fold variability under group-wise splitting. Moreover, labels differ across corpora (speaker-level pathology in TORGO vs. transient intraoperative impairment), complicating transfer. Future work should prioritize patient-level calibration and larger multi-center clinical cohorts with standardized protocols.

## Conclusion

We proposed a dual-view EfficientNet-B0 framework that fuses two complementary encodings of each log-mel spectrogram (NPY amplitude view and PNG colormapped view) for short-segment dysarthria detection in awake surgery. In intra-corpus evaluation, fusion improved discrimination on TORGO, with gated fusion achieving the best performance. On the intraoperative DATABRASE corpus, results were highly variable across patient folds: PNG inputs performed best on raw recordings, whereas Demucs denoising enabled attention-based fusion to achieve the most balanced UAR performance. In cross-corpus transfer (TORGO→DATABRASE), all methods exhibited substantial performance degradation, confirming a severe domain shift between controlled English recordings and French intraoperative speech. This finding also highlights the need to explicitly evaluate cross-language robustness in future intraoperative speech-monitoring systems. Future work should focus on domain-robust learning, including self-supervised and domain-adaptive training, as well as patient-level calibration to reduce false positives in clinical conditions.

## Data Availability

The TORGO corpus is publicly available at https://www.cs.toronto.edu/~complingweb/data/TORGO/torgo.html. Access to the DATABRASE intraoperative recordings is restricted due to patient confidentiality and can be considered upon reasonable request and appropriate ethical approval.
